# A Cross-Sectional Study Comparing the Efficacy of Various Growth Charts in Evaluating the Incidences of Small for Gestational Age and Large for Gestational Age at Birth Among Liveborn Neonates Delivered at a Tertiary Teaching Hospital

**DOI:** 10.7759/cureus.38058

**Published:** 2023-04-24

**Authors:** Rufus R Singamala, Preethi Subramanian, Sudharshan R Chitgupikar

**Affiliations:** 1 Pediatrics, Government Medical College Suryapet, Suryapet, IND; 2 Pediatrics, Mediciti Institute of Medical Sciences, Hyderabad, IND

**Keywords:** kandraju chart, intergrowth chart, fenton 2013, birth weight, small for gestational age (sga), large for gestational age (lga), growth charts

## Abstract

Background and objective

Growth charts are important in monitoring the growth of neonates. The growth of Indian fetuses is understood to be different from the Western population due to multiple factors. In this study, we aimed to analyze the utility of the application of various growth charts in evaluating the birth weights of liveborn neonates at a tertiary teaching hospital.

Methodology

A total of 729 liveborn neonates between 24 to 42 weeks of gestation delivered at the study institute during the study period were included. Birth weights were plotted on three growth charts - Fenton 2013, INTERGROWTH-21st (IG-21), and Kandraju et al. chart - and classified as small for gestational age (SGA), or appropriate for gestational age (AGA), or large for gestational age (LGA) according to the respective centiles and sex. The incidences of SGA and LGA were calculated with respect to various charts and compared. Statistical analysis was done using the McNemar Chi-square test for paired categorical variables. Cohen’s kappa (K) was used to analyze the concordance between the growth charts. A p-value <0.005 was considered statistically significant.

Results

Among 668 term neonates, the number of neonates classified as SGA was 313 (46.86%), 236 (35.33%), and 219 (32.78%) according to Fenton 2013, IG-21, and Kandraju et al. chart respectively. The difference in incidences of SGA between Fenton 2013 and IG-21 for term neonates was significant (p=0.0001). The difference between incidences of SGA among term neonates according to Fenton 2013 and Kandraju et al. and IG-21 vs. Kandraju et al. was significant (p=0.0001). Among 61 preterm neonates, the number of neonates classified as SGA was 15, 11, and five according to Fenton 2013, IG-21, and Kandraju et al. respectively. There was no statistically significant difference between the three charts. Among 729 neonates, the number of neonates classified as LGA was 10 (1.37%), 22 (3.02%), and 32 (4.39%) according to Fenton 2013, IG-21, and Kandraju, et al. respectively. The difference in incidences of LGA between Fenton 2013 and IG-21 was significant (p=0.0015). The difference in incidences of LGA between Fenton 2013 and Kandraju et al. was significant (p=0.0001). The difference in incidences of LGA between IG-21 and Kandraju et al. was also significant (p=0.0044).

Conclusion

Fenton 2013, IG-21, and Kandraju et al. growth charts vary significantly in detecting the incidence of SGA and LGA among term neonates. Among term neonates, IG-21 and Kandraju et al. growth charts are comparable in terms of the estimation of SGA. The Fenton 2013 growth chart showed a higher incidence of SGA among term neonates. The incidence of LGA was highest according to Kandraju et al. growth chart and least according to Fenton 2013. Among preterm neonates, the incidence of SGA as per birth weight was comparable across the three growth charts.

## Introduction

Birth weight is the single most important predictor of growth potential. Growth of the fetus varies not only among individuals but also across populations due to differences in ethnicity, geography, socioeconomic status, and environmental, nutritional, and hereditary factors [1-3]. Most of the available growth charts are based on the Western population. There are different types of growth charts such as reference growth charts and standard growth charts. Growth reference charts describe how neonates actually grow and are used to establish whether or not their measurements are typical of the reference group. Growth standard charts are essentially the same as growth reference charts except that the underlying reference sample is selected on health grounds. They pertain to a healthy pattern of growth. The standard charts show how the neonates ought to grow rather than how they do grow [4]. INTERGROWTH-21st (IG-21) is a standard growth chart developed based on data obtained from fetuses and neonates (26-32 weeks of gestational age) across eight countries from different regions of the world [5]. Fenton 2013 [6] is a reference growth chart published in 2013 for preterm neonates from 22 weeks to 50 weeks of gestational age. Kandraju et al. chart [7] is a regional growth chart based on the South Indian population published in 2011 based on the data from a level III maternity and newborn hospital-based study involving 31,391 neonates on birth weight across 24 to 42 weeks of gestational age.

Indian neonates are among the smallest in the world, especially compared to the neonatal population from developed countries [8]. Hence, when Indian neonates are classified using the Western-based charts, there is a risk of overestimation of small for gestational age (SGA) neonates as many appropriate for age (AGA) neonates could get classified as SGA and underestimation of large for gestational age (LGA) neonates. An accurate diagnosis of SGA and LGA is essential as the implications of this classification on the health of infants during the perinatal period and in the future are significant. The SGA and LGA neonates are at risk of developing metabolic syndromes later in life due to various pathophysiological changes that occur in utero [9-11]. In this study, we aimed to plot the birth weight of neonates using the centiles provided by the above three growth charts and to compare the incidences of SGA and LGA among infants as per the data from these charts.

## Materials and methods

This was a cross-sectional study conducted at a tertiary care teaching hospital in Hyderabad, Telangana, South India. A total of 729 liveborn neonates between 24 to 42 weeks of gestation were included in the study. The study was conducted from January 2020 to June 2021, after obtaining Institutional Ethical Committee (IEC) clearance (Mediciti Ethics Committee: dt:10/10/2019/S.No:11).

The sample size required was calculated based on the prevalence of SGA and LGA neonates as documented in previous studies. The prevalence of SGA was 46.9% based on the data gathered from previous birth cohort studies [12] and the prevalence of LGA was 9.4% [13]. Based on this data, the sample size was calculated with the formula n={z^2* p*(1-p)}/d^2, where n is the sample size, z is the confidence interval (which was taken as 95%), p is the prevalence observed in the population, and d is the margin of error taken as %. The sample size was 383 based on prevalence for SGA neonates and 131 for LGA neonates. With a 10% margin of error, the sample size required was determined to be 421.

The inclusion criteria were as follows: all liveborn neonates between 24 and 42 weeks of gestation delivered at the study institute during the study period. The exclusion criteria were as follows: intrauterine deaths and stillbirths, neonates with gross congenital anomalies, multiple pregnancies (twins, triplets, etc.), outborn neonates, and all liveborn neonates born after 42 weeks of gestational age. All the neonates who fulfilled the inclusion and exclusion criteria were consecutively sampled. Owing to the coronavirus disease 2019 (COVID-19) pandemic, the number of hospital deliveries was low during the study period, and a total of 729 neonates were finally enrolled.

Informed written consent was obtained from the parents of all neonates included in the study. A preformatted proforma was used to collect the relevant maternal and neonatal data. Maternal details such as age at conception, last menstrual period (LMP), stature, and socioeconomic status based on the Kuppuswamy scale [14], comorbidities present during pregnancy such as hypertension, diabetes mellitus, hypothyroidism, ultrasonogram reports during pregnancy, and drug intake if any were documented. For neonates, date and time of birth, birth weight, head circumference, and length along with general examination were documented. Birth weight was recorded within 10 minutes of birth after stabilizing the neonate. The weight was measured on a digital weighing machine with a variability of ±10 grams. The neonate was weighed three times and an average of the three readings was taken as the final birth weight. The other measurements were taken within 24 hours of birth. The gestational age was calculated as per LMP or first-trimester ultrasound (dating scan) when LMP was not available/reliable. The birth weight of all the neonates was plotted on Fenton 2013, IG-21, and Kandraju et al. growth charts. They were classified as SGA, AGA, and LGA according to the respective centiles and compared. SGA was defined as a weight below the 10th percentile for gestational age and gender as per the population growth charts. AGA was defined as the weight between the 10th percentile and 90th percentile for gestational age and gender as per the population growth charts. LGA was defined as weight above the 90th percentile for gestational age and gender as per the population growth charts [15]. The incidences of SGA and LGA were calculated accordingly with respect to various charts.

Statistical analysis

Data were entered into a Microsoft Excel sheet and analyzed using IBM SPSS Statistics version 22.0 (IBM Corp., Armonk, NY). Descriptive statistics were expressed as mean and standard deviation (SD) for continuous variables and as proportions and percentages for categorical variables. Inferential statistical analysis was done using McNemar's Chi-square test for paired categorical variables. Cohen’s kappa (K) [16] statistic was used to analyze the concordance between the growth charts. A p-value <0.005 was considered statistically significant.

## Results

In this cross-sectional study, out of 745 neonates, 16 neonates were excluded (seven twins, six intrauterine deaths, and three congenital anomalies), and 729 neonates (388 males) were included. Of them, 61 were preterm neonates and 668 were term neonates. The demographic characteristics of mothers and their comorbidities are listed in Table 1.

**Table 1:** Demographic characteristics of mothers and their comorbidities SD: standard deviation

Variables	Values
Mothers' age, years, mean ±SD	23.931 ±3.366
Mothers' height, cm, mean ±SD	156.57 ±4.013
Number of mothers with diabetes mellitus complicating pregnancy	51
Number of mothers with gestational diabetes mellitus	47
Number of mothers with overt diabetes	4
Number of mothers with hypertension	61
Number of mothers with gestational hypertension	31
Number of mothers with preeclampsia	21
Number of mothers with eclampsia	9
Number of mothers with hypothyroidism	29
Number of mothers with diabetes mellitus and hypertension	5

Based on weight centiles of IG-21 charts, 11 neonates were SGA among the neonates born to 51 mothers with diabetes complicating the pregnancy; 29 neonates were SGA among the neonates born to 56 mothers with hypertension. Eight neonates were SGA among the neonates born to 29 mothers with hypothyroidism.

Among the 729 neonates, the classification of neonates as SGA, AGA, and LGA according to the three charts is presented in Table 2.

**Table 2:** Distribution of all neonates according to their intrauterine growth status by various growth charts IG-21: INTERGROWTH-21st

Categories	Classification of neonates by Fenton 2013 chart [6], n (%)	Classification of neonates by IG-21 chart [5], n (%)	Classification of neonates by Kandraju et al. chart [7], n (%)
Small for gestational age	328 (44.99)	247 (33.88)	224 (30.73)
Appropriate for gestational age	391 (53.64)	460 (63.10)	473 (64.88)
Large for gestational age	10 (1.37)	22 (3.02)	32 (4.39)
Total	729	729	729

The incidence of SGA according to Fenton 2013 (44.99%) was higher compared to the incidence of SGA according to IG-21 (33.88%). The difference in incidences of SGA between IG-21 and Fenton 2013 (n=81) was significant (p<0.00001, McNemar's Chi-square test); 81 neonates classified as SGA according to Fenton 2013 growth chart were classified as AGA according to IG-21. Cohen’s kappa coefficient (K) to evaluate the agreement between the charts was 0.749 (CI: 0.703-0.796), which indicates substantial agreement. The growth charts were different but comparable.

The difference between incidences of SGA according to IG-21 (33.88%) and Kandraju et al. (30.73%) was significant (p=0.0001); 224 neonates classified as SGA according to Kandraju et al. were also classified as SGA by IG-21 chart; 23 neonates classified as SGA according to IG-21 were classified as AGA according to Kandraju et al. chart. The agreement between these two growth charts was almost perfect with Cohen’s kappa value of 0.907 (CI: 0.876-0.938).

The difference between incidences of SGA according to Fenton 2013 (44.99%) and Kandraju et al. (30.73%) was significant (p=0.0001); 104 neonates classified as SGA according to Fenton 2013 was classified as AGA according to Kandraju et al. The agreement between the two charts was substantial with a K value of 0.686 (CI: 0.635-0.736).

Among preterm neonates (n=61), the classification into SGA, AGA, and LGA according to the various charts is shown in Table 3.

**Table 3:** Distribution of preterm neonates according to their intrauterine growth status by various growth charts IG-21: INTERGROWTH-21st

Categories	Classification of neonates by Fenton 2013 chart [6], n (%)	Classification of neonates by IG-21 chart [5], n (%)	Classification of neonates by Kandraju et al. chart [7], n (%)
Small for gestational age	15 (24.59)	11 (18.03)	5 (8.20)
Appropriate for gestational age	41 (67.21)	43 (70.49)	44 (72.13)
Large for gestational age	5 (8.20)	7 (11.48)	12 (19.61)
Total	61	61	61

The difference between incidences of SGA according to Fenton 2013 (24.59%) and IG-21 (18.03%) was not significant (p=0.1336). The four preterm neonates classified as SGA by Fenton 2013 were classified as AGA by IG-21. The agreement between these charts was substantial (K=0.792; CI: 0.634-0.950). The difference between incidences of SGA according to IG-21 (18.03%) and Kandraju et al. (8.20%) was not significant (p=0.0412). Six preterm neonates classified as SGA by IG-21 were classified as AGA by Kandraju et al. The agreement between these charts was moderate (K=0.603; CI: 0.396-0.810). The difference between incidences of SGA according to Fenton 2013 (24.59%) and Kandraju et al. (8.20%) was significant (p=0.0044). Ten preterm neonates classified as SGA by Fenton 2013 were classified as AGA by Kandraju et al. The agreement between the two charts was fair (K=0.366; CI: 0.298-0.434).

The classification of term neonates into SGA, AGA, and LGA according to the various charts is presented in Table 4.

**Table 4:** Distribution of term neonates according to their intrauterine growth status as per various charts IG-21: INTERGROWTH-21st

Categories	Classification of neonates by Fenton 2013 chart [6], n (%)	Classification of neonates by Intergrowth -21 (IG-21) chart [5] n (%)	Classification of neonates by Kandraju et al. chart [7] n (%)
Small for gestational age	313 (46.86)	236 (35.33)	219 (32.78)
Appropriate for gestational age	350 (52.39)	417 (62.43)	429 (64.23)
Large for gestational age	5 (0.75)	15 (2.24)	20 (2.99)
Total	668	668	668

Among 313 term neonates classified as SGA according to Fenton 2013, 77 (24.6%) were classified as AGA according to the IG-21 growth chart. The difference between incidences of SGA according to Fenton 2013 (46.86%) and IG-21 (35.33%) for term neonates was significant (p=0.0001). The agreement between the charts was substantial (K=0.743; CI: 0.694-0.792).

Among 236 term neonates classified as SGA according to IG-21, 219 were classified as SGA and 17 as AGA according to Kandraju et al. The difference was statistically significant (p=0.0001). The agreement between the curves was almost perfect (K=0.932; CI: 0.904-0.960).

Among 313 term neonates classified as SGA according to Fenton 2013, 219 were classified as SGA and 94 as AGA according to Kandraju et al. The difference between incidences of SGA according to Fenton 2013 and Kandraju et al. was significant (p=0.0001). The agreement was substantial (K=0.680; CI: 0.627-0.732).

Among 729 neonates, the number of neonates classified as LGA was 10 (1.37%), 22 (3.02%), and 32 (4.39%) according to Fenton 2013, IG-21, and Kandraju et al. respectively. The difference in incidences of LGA between Fenton 2013 and IG-21 was significant (p=0.0015). The difference in incidences of LGA between IG-21 and Kandraju et al. was significant (p=0.0044). The difference in incidences of LGA between Fenton 2013 and Kandraju et al. was also significant (p=0.0001).

A comparison of the various charts with each other and their agreement by Cohen's K [17] is tabulated in Table 5.

**Table 5:** Cohen’s kappa (K)* values based on the comparison of charts with each other *[15] IG-21: INTERGROWTH-21st

Comparison of charts	Cohen’s kappa value for all neonates	Cohen’s kappa value for preterm neonates	Cohen’s kappa value for term neonates
Fenton 2013 vs. IG-21	0.749	0.792	0.743
IG-21 vs. Kandraju et al.	0.907	0.603	0.932
Fenton 2013 vs. Kandraju et al.	0.686	0.366	0.680

## Discussion

The number of neonates classified as SGA was 328, 247, and 224 according to Fenton 2013, IG-21, and Kandraju et al. respectively. The incidence of SGA according to Fenton 2013 (44.99%) was higher compared to the incidence of SGA according to IG-21 (33.88%). This could be attributed to the fact that the Fenton 2013 growth charts are based on the data obtained from neonates born in affluent countries where the growth potential itself is high and mothers have better socioeconomic status. The incidence of SGA according to Kandraju et al. (30.73%) was relatively lower than IG-21 (33.88%). Kandraju et al. growth charts are based on the South Indian population whose standards for the centiles seem to be lower than IG-21. The growth standards of Kandraju et al. are lower than IG-21, which in turn are lower than the Fenton 2013 growth chart. This indicates that South Indian neonates are smaller and lighter when compared to the Western population (Fenton 2013) and the population on which IG-21 was originally based.

Our findings are similar to those of a cross-sectional study done in 2019 by Tenório et al. [17] in Brazil. The authors observed that among 344 neonates between 33-43 weeks of gestational age (20 preterm and four post-term), the incidence of SGA was lower according to IG-21 (4.9%) as compared to Fenton 2013 (16.9%) and Alexander et al. growth chart (18.6%). The study observed that the difference in incidences of SGA between IG-21 and Fenton 2013 and between IG-21 and Alexander et al. charts was significant with a p-value <0.001 for both comparisons and K of 0.5625 and 0.5581 respectively. Similarly, Jakubowski et al. [18] observed significant differences between Fenton, IG-21, and WHO growth charts for the detection of SGA and LGA among 8,608 neonates from Poland, across 24-40 weeks of gestational age.

Among the 61 preterm neonates in this study, only four neonates who were classified as SGA according to the Fenton 2013 growth chart were classified as AGA according to the IG-21 growth chart. The difference between incidences of SGA according to Fenton 2013 and IG-21 was not significant. This shows that preterm growth centiles of Fenton 2013 and IG-21 seem to have good concordance with each other. A retrospective study published in 2020 by Lebrao et al. [19] in Brazil among 173 preterm neonates (birth weight of less than 1500 grams and gestational age between 26 to 33 weeks) suggested that Fenton 2013 and IG-21 were similar in terms of classification of birth weight for preterm neonates. They observed a similar incidence of SGA (35.2% in Fenton vs. 39.2 in IG-21) with Cramer’s test V score of 0.533, indicating very strong agreement. However, a study by Patel et al. [20] from India among 301 preterm neonates in 2021 concluded that on comparison of Fenton and IG-21 charts for preterm neonates, the agreement between the charts was poor as the Fenton chart identified more neonates to have growth restriction compared to IG-21.

Among the 668 term neonates in our study, the number of neonates classified as SGA were 313, 236, and 219 according to Fenton 2013, IG-21, and Kandraju et al. respectively. This significant difference in term neonates as compared to preterm neonates could be because the Fenton 2013 curves do not represent the actual growth beyond 36 weeks of gestational age. It was mathematically constructed using the cross-sectional data of preterm neonates between 24 to 36 weeks. The accuracy of these curves for term neonates is questionable. According to a retrospective study of 2849 neonates done in Brazil by Barreto et al. [21] in 2020, the incidence of SGA neonates between 34 to 42 weeks of gestation was higher according to Fenton 2013 (13%) than IG-21 (8.7%). In their study, among 2628 term neonates, the difference in incidences of SGA between Fenton 2013 (13.1%) and IG-21 (8.5%) was significant (p<0.001, K=0.532). The authors concluded that the incidence of SGA neonates was significantly higher as per the Fenton 2013 curve, with a greater agreement between the Fenton 2013 and IG-21 curves among preterms when compared to full-term neonates.

In our study, the difference in incidences of LGA between Fenton 2013 and IG-21 was significant (p=0.0015). In the study by Tenório et al. [17] among 344 neonates, the incidence of LGA was 9.9% according to IG-21 and 9.6% according to Fenton 2013. The difference in incidences of LGA between Fenton 2013 and IG-21 was significant (p<0.001).

A study published by Tuzun et al. [22] in 2017 involving 248 preterm neonates showed the incidence of LGA as 6% according to both Fenton 2013 and IG-21. A study by Prakash et al. [23] from South India involving 2507 neonates observed discrepancies in the detection of LGA between Fenton 2013 (1.4%) and IG-21 (5.5%). These differences among the international charts that are generally used for labeling neonates as SGA and LGA are important and should not be overlooked. This study highlights the need for conducting large-scale population-specific studies on a national level that will help construct growth charts targeting our Indian neonates.

Limitations

This study was based on a small sample size with a relatively lower number of preterm neonates. Moreover, the study did not look into the causative factors (maternal, placental, or fetal) for SGA and LGA.

## Conclusions

The Fenton 2013, IG-21, and Kandraju et al. growth charts vary significantly in detecting the incidence of SGA and LGA among term neonates. Among term neonates, IG-21 and Kandraju et al. growth charts are comparable for the estimation of SGA. The Fenton 2013 growth chart showed a higher incidence of SGA among term neonates. The incidence of LGA was highest according to Kandraju et al. growth chart and least according to the Fenton 2013 growth chart. Among preterm neonates, the incidence of SGA using birth weight was comparable across the three growth charts. It could be understood that while population-specific growth charts might be required for term neonates, all three charts are comparable and could be applied among preterm neonates.
